# A Two-Component-System-Governed Regulon That Includes a β-Lactamase Gene is Responsive to Cell Envelope Disturbance

**DOI:** 10.1128/mbio.01749-22

**Published:** 2022-08-15

**Authors:** Dongju Lee, Jongwook Park, Hyojeong Yi, Kwang-Hwi Cho, Heenam Stanley Kim

**Affiliations:** a Division of Biosystems and Biomedical Sciences, College of Health Sciences, Korea University, Seoul, South Korea; b School of Systems Biomedical Science and Research Center for Integrative Basic Science, Soongsil University, Seoul, South Korea; Louis Stokes Veterans Affairs Medical Center

**Keywords:** *Burkholderia*, β-lactamase, PenL, envelope stress, two-component system, BesRS

## Abstract

β-Lactamase production facilitates bacterial survival in nature and affects many infection therapies. However, much of its regulation remains unexplored. We used a genetics-based approach to identify a two-component system (TCS) present in a strain of Burkholderia thailandensis essential for the regulated expression of a class A β-lactamase gene, *penL*, by sensing subtle envelope disturbance caused by β-lactams, polymyxin B, or other chemical agents. The genes encoding stress responses and resistance to various antibiotics were coregulated, as were the catabolic genes that enabled the B. thailandensis strain to grow on penicillin G or phenylacetate, a degradation product of penicillin G. This regulon has likely evolved to facilitate bacterial survival in the soil microbiome that contains a multitude of antibiotic producers. Practically, this regulatory system makes this TCS, which we named BesRS, an excellent drug target for the purpose of increasing antibiotic efficacy in combination therapies for *Burkholderia* infections.

## INTRODUCTION

In Gram-negative bacteria, expression levels of β-lactamases are usually low but are inducible when the cells are exposed to β-lactams (1–3). β-Lactamase induction mechanisms linked with the cell wall metabolism have been found in various bacteria, in which, cell wall fragments, such as muropeptides, liberated to the periplasm due to the cell wall hydrolysis by β-lactams, act as inducers (1, 3, 4). These inducers are transported into the cytoplasm where they bind to an AmpR-type regulator turning it into an activator, which then can activate a β-lactamase gene, typically *ampC* (1–5). Different β-lactamase gene regulatory pathways involving two-component systems (TCSs) have been reported (3, 6–8). The exact mechanisms involved in triggering these TCSs need to be explored. However, at least for the CpxA sensor kinase from Klebsiella aerogenes, cell wall fragments likely are the inducers for *ampC* expression (9). A different mechanism that does not involve the cell wall metabolism has been recently discovered in the marine bacterium Vibrio parahaemolyticus (10). This regulation mechanism involves a sensor kinase of a TCS directly detecting β-lactam molecules, instead of cell wall fragments, and binding to them leading to β-lactamase gene induction (10).

Chromosomally encoded PenI-type (also called, PenA-type) class A β-lactamase protects Burkholderia pseudomallei and Burkholderia mallei from β-lactam antibiotics such as amoxicillin (11–14). B. pseudomallei and B. mallei are the causative agents of melioidosis and glanders, respectively, and are serious human and animal health hazards in endemic areas throughout the world (15, 16). Both species are listed as category B potential biowarfare agents by the U.S. Centers for Disease Control and Prevention (CDC). PenI-type β-lactamase has been actively studied in the nonpathogenic bacterium Burkholderia thailandensis (17–19), which is a safe laboratory model for studying B. pseudomallei and B. mallei due to its close relatedness to these species (20). The PenL enzyme in B. thailandensis (BTH_RS07435, old_locus_tag: BTH_II1450, formerly called PenA) shares 89% of amino acid identity with its orthologs in B. pseudomallei and B. mallei, and the genes encoding these enzymes are present in the highly syntenic chromosome 2 in each species (20).

The PenI-type β-lactamase can transition into an extended-spectrum β-lactamase (ESBL) by acquiring a mutation that enables it to hydrolyze ceftazidime, while losing its activity toward its original β-lactam substrates (17–19). Ceftazidime is most frequently used as first-line therapy for infections with *Burkholderia* spp. In the presence of ceftazidime (4 to 7 μg/mL), various mutations (mostly single-amino-acid substitutions, but also small deletions or duplications) can occur in the B. thailandensis *penL* gene, conferring increased hydrolytic activity of the enzyme toward ceftazidime and other third-generation cephalosporins (MICs for ceftazidime: 6 to 80 μg/mL) (17–19).

Intriguingly, B. thailandensis strains carrying an ESBL-coding *penL* gene can further evolve to survive at even higher ceftazidime levels by acquiring a mutation in the *penL* promoter (17). A single-nucleotide substitution of G to A in the –10 sequence increases its similarity to the consensus sequence, making a stronger promoter with increased *penL* expression (17). The same promoter mutation has been selected for in B. pseudomallei clinical isolates with wild-type or ESBL-coding *penA* (the *penL* ortholog) (13), suggesting that high β-lactamase expression can significantly interfere with antibiotic regimens. Despite the clinical significance of an increased expression of this β-lactamase gene, its regulation is largely unknown.

In this study, we report a novel regulatory mechanism of the β-lactamase gene, *penL*, which is induced by a TCS in response to cell envelope disturbance. We show that this gene is part of a regulon involved in multiple antibiotic resistances, stress responses, and the catabolism of β-lactam-derived products. This regulon may have evolved to facilitate survival of *Burkholderia* spp. in the soil environment containing a multitude of antibiotics, including β-lactams (21–23).

## RESULTS

### Discovery of a TCS associated with β-lactam resistance.

**FIG 1:**
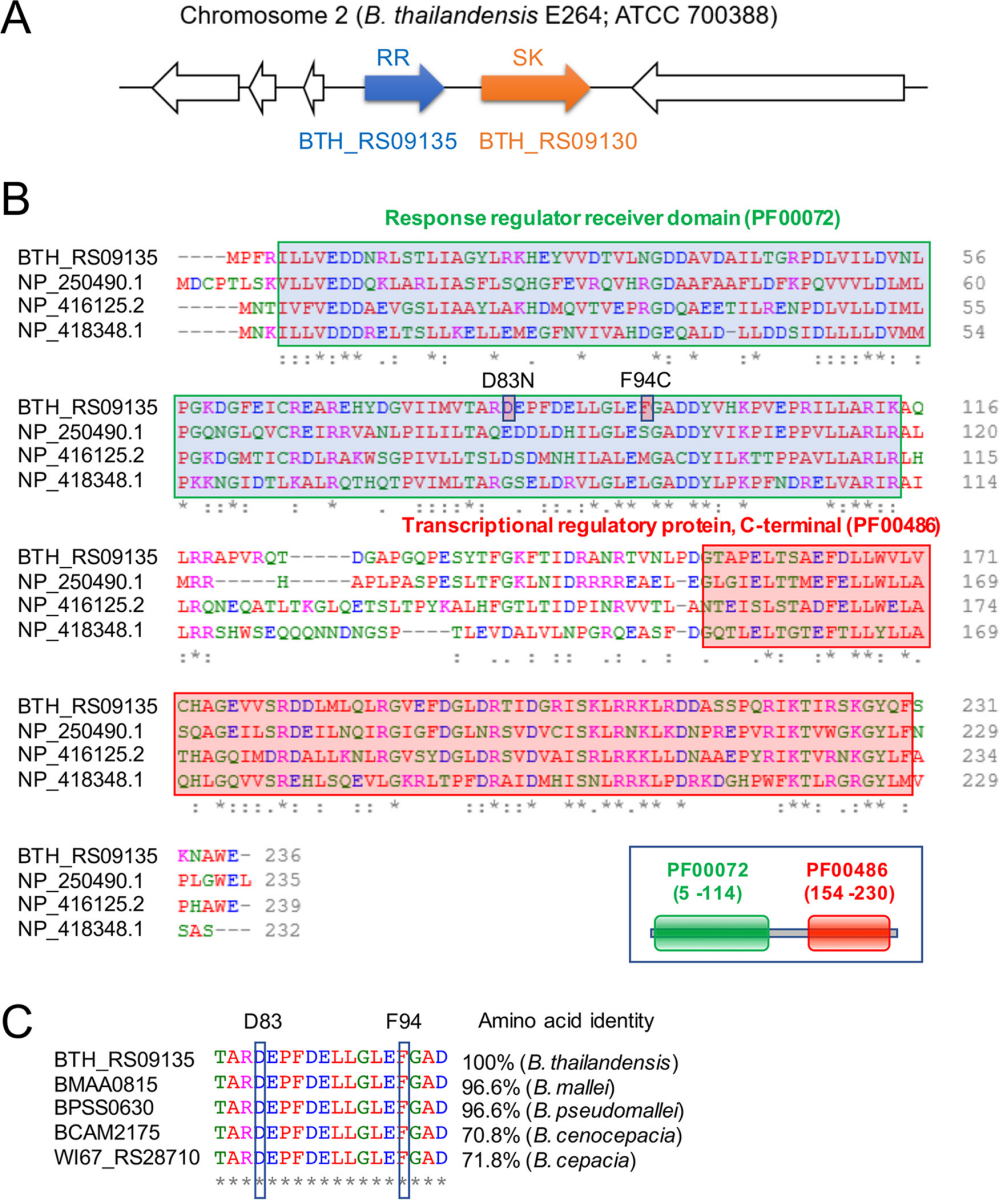
Two mutations in a two-component system (TCS) conferring an increased ceftazidime resistance in ESBL-producing Burkholderia thailandensis. (A) Organization of genes encoding the TCS in the chromosome 2. RR, response regulator; SK, sensor kinase. (B) The RR of a TCS with the mutations. The amino acid sequence of the RR encoded by the gene BTH_RS09135 in B. thailandensis E264 is aligned with its close homologs in other bacteria (NP_250490.1, ParR from Pseudomonas aeruginosa; NP_416125.2, RstA from E. coli; NP_418348.1, CpxR from E. coli). The amino acid residues D and F substituted in mutants as D83N and F94C, respectively, are denoted in small boxes. Two Pfam domains, PF00072 and PF00486, are denoted in green and red boxes on the sequences, respectively. (C) Conservation of the two amino acid residues D83 and F94 among the RR orthologs in *Burkholderia* spp.

To investigate the mechanism underlying the β-lactamase gene expression in *Burkholderia* spp., we took a genetic approach performing an antibiotic selection using the B. thailandensis strain E264 that harbors a gene expressing ESBL-PenL, which has a mutation of either Asp179Asn or Glu166Lys in PenL, extending the substate spectrum of the enzyme to include ceftazidime (17). The strains having a mutation of Asp179Asn and Glu166Lys in PenL, which are called W35 and W36, respectively, were challenged with a lethal dose of ceftazidime (100 μg/mL). Most mutants (≈ 90%) that survived the selection had the same *penL* promoter mutation we reported in a previous study (G to A in the –10 sequence) (17). However, we also found a different type of mutants (≈ 10%) with a mutation in a gene (BTH_RS09135) encoding a response regulator (RR) of a TCS that was not previously characterized or named (Fig. 1A). These mutants had a missense mutation of either Asp83Asn (a codon GAC changed to AAC) or Phe94Cys (a codon TTC changed to TGC) in the RR (Fig. 1B) and the strains containing these mutations were called W35F2 and W36F2, respectively. Both mutations were located at the response regulator receiver domain PF00072 in the RR, which is closely related to its homologs in many pathogens, including Pseudomonas aeruginosa and Escherichia coli (Fig. 1B). Importantly, the amino acid residues Asp83 and Phe94 were found to be highly conserved in a majority of *Burkholderia* pathogens, suggesting they may play a pivotal role in RR function and similar missense mutations may occur causing similar effects in these pathogens (Fig. 1C). A sensor kinase (SK)-coding gene (BTH_RS09130) was found immediately downstream of this mutated gene, similar to that seen in many gene pairs encoding a TCS (24, 25) (Fig. 1A).

**FIG 2:**
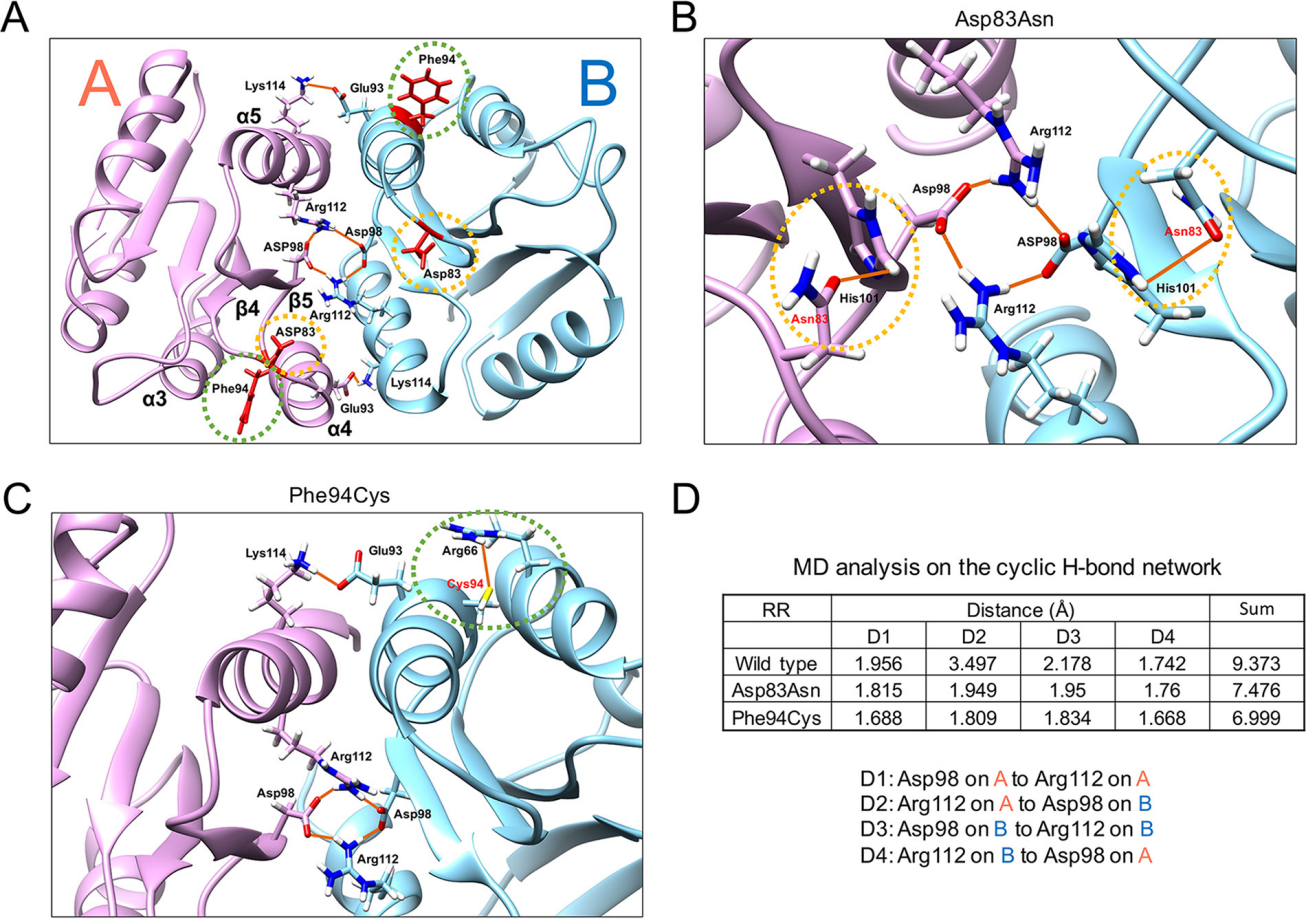
The 3D structure of the response regulator receiver domain (PF00072) of the RR. (A) The simulated 3D structure of the response regulator receiver domains in their dimeric form. Two peptides are labeled A and B and colored differently. The original amino acid residues that are substituted in mutants are denoted by orange (Asp83Asn) and green (Phe94Cys) dotted circles. (B) The Asp83Asn mutant. Newly formed hydrogen bond involving Asn83 is denoted by a dotted orange circle. (C) The Phe94Cys mutant. Newly formed hydrogen bond involving Cys94 is denoted by a dotted green circle. (D) Molecular dynamics analysis. The average distances of the cyclic H-bonds are compared between the wild-type and the mutant RRs.

TCSs allow bacteria to mount appropriate adaptive responses when specific environmental signals are perceived (24, 25). TCSs are signal transduction systems in which an SK detects specific environmental signals and becomes autophosphorylated. The phosphate is transferred to its cognate RR, which is then activated to form a homodimer; this dimeric RR regulates the transcription of genes under its control (24, 25). In the case of the RR encoded by the gene BTH_RS09135, two intermolecular hydrogen bonds appear to contribute to the dimerization of RR monomers: the first between residues Glu93 and Lys114 and the second between residues Arg112 and Asp98. Together, these interactions form a cyclic hydrogen bond network (Fig. 2A). The two mutations, Asp83Asn and Phe94Cys, are located close to the interface of the monomers (Fig. 2A). The Asn residue replacing Asp83 can form a new hydrogen bond with His101, increasing the intramolecular stability of the RR (Fig. 2B). Similarly, the new Cys94 can form a hydrogen bond with Arg66, stabilizing the interaction between two α chains (α3 and α4) (Fig. 2C). Such increases in intramolecular stability may have resemblance to the alteration of intramolecular interactions mediated by phosphorylation, which has been implicated in the dimerization process (26). To determine if the mutation-mediated internal stabilization facilitates RR dimerization, we performed molecular dynamics simulations of the wild-type and the two mutant RRs. Average distance for the cyclic hydrogen bond network in the mutant RRs was shorter than that in the wild-type RR (Fig. 2D), suggesting the mutant RRs may form a highly stabilized dimer, perhaps similar to that formed by phosphorylation effect, which facilitates the binding of the dimer to target DNA (27).

By comparing the RR and the SK of this uncharacterized TCS from B. thailandensis with those of known TCSs, we found high amino acid sequence matches with RstAB (with RstA: 45.42% and RstB: 35.8%), EnvZ/OmpR (with EnvZ: 31.18% and OmpR: 44.16%), CpxAR (with CpxA: 32.03% and CpxR: 37.89%), and BaeSR (with BaeS: 29.29% and BaeR: 38.63%), which are all from E. coli. RstAB has been shown to play key roles in multiple virulence-associated processes in enterohemorrhagic E. coli O157:H7 (28) and in Photobacterium damselae subsp. *damselae* (29). In *Photobacterium*, mutations in RstAB abolished bacterial resistance to benzylpenicillin, suggesting the involvement of RstAB in the regulation of the β-lactam resistance (29). Notably, EnvZ/OmpR, BaeSR, and CpxAR have been shown to respond to disruptions in the bacterial envelope integrity, which is often linked to the induction of virulence, various cellular processes, and resistance to antimicrobial agents (30–33). For example, BaeSR has been shown to induce AdeABC efflux pump conferring tigecycline resistance in Acinetobacter baumannii (33). A study showed CpxAR activated the *ampC* β-lactamase gene when cell wall metabolism was disrupted by interactions with imipenem (9). Altogether, accumulating evidence suggests that TCSs play a key role in regulating the antimicrobial resistance in various bacteria.

### Mutations in the RR resulted in an increased β-lactamase expression.

To determine the extent to which RR mutations affect antibiotic resistance in B. thailandensis, we profiled the MICs of the two mutant strains, W35F2 and W36F2, for various β-lactams and non-β-lactams (Table 1). Like ceftazidime, the MICs for cefotaxime, another third-generation cephalosporin, were severalfold higher for the mutants than for their wild-type parental strains (Table 1). However, the MICs for various antibiotics that are not third-generation cephalosporins, such as ampicillin, meropenem, ciprofloxacin, kanamycin, and tetracycline, were not significantly different for the mutants from those of their parental strains (Table 1). Notably, ceftazidime and cefotaxime are the only digestible substrates for ESBL-PenLs in this list of tested antibiotics. Therefore, these MIC profiles correspond to the substrate spectrum of the ESBL-PenL, suggesting the RR mutations may confer increased ESBL-PenL activity. Consistently, disruption of the ESBL-coding *penL* gene in the mutant strain W36F2 abolished the increased resistance to both ceftazidime and cefotaxime (Table 1).

**TABLE 1:** MICs (μg/mL) of strains for various antibiotics

Strain^*a*^	Description	AMP^*b*^	CAZ	CTX	MER	CIP	KAN	TET
ESBL-PenL background								
W35	E264 (PenL with Asp179Asn)	8	32	4	1	2	128	8
W35F2	W36 (RR with Asp83Asn)	32	256	32	2	2	128	8
W36	E264 (PenL with Glu166Lys)	4	64	4	1	2	128	8
W36F2	W36 (RR with Phe94Cys)	4	192	16	2	2	128	8
W36F2ΔPenL*	36F2 (ESBL-coding *penL*::tet^R^)	6	0.9	1.3	1	1.8	128	ND^*c*^
W36F2ΔRR*	36F2 (rr*::tet^R^)	6.7	15.7	2	1.2	1.8	128	ND
W36F2ΔSK	36F2 (*sk*::tet^R^)	6.7	32	1.7	1.2	1.8	128	ND
W36F2ΔRR*(p-RR)	36F2Δrr* (pRK415K::*rr-sk*)	6.5	136	8	1.6	2.5	ND	ND
W36F2ΔRR*(p-RR*)	36F2Δrr* (pRK415K::rr*-sk)	6.7	256	16	2	2.7	ND	ND
WT-PenL background								
E264	Wild-type	32	1	8	1.2	2.2	128	8
E264ΔRR	E264 (*rr*::tet^R^)	18.7	1	4	1.2	2.2	128	ND^*b*^
E264ΔSK	E264 (*sk*::tet^R^)	18.7	1	4	1.2	2.2	128	ND
E264ΔRR(p-RR)	E264 (pRK415K::*rr*)	149.3	4	32	2	3.7	ND	ND
E264ΔRR(p-RR*)	E264 (pRK415K::rr*)	234.7	6.3	64	2	2.7	ND	ND

a Abbreviations in strain: RR, the response regulator encoded by BTH_RS09135; PenL*, PenL with Glu166Lys; tet^R^, the tet^R^ cassette (17); RR*, the response regulator with a mutation; *rr*, the gene (BTH_RS09135) encoding the response regulator; rr*, *rr* with a mutation; SK, the sensor kinase; *sk*, the gene (BTH_RS09130) encoding the sensor kinase; *rr*-*sk*, the operon encoding the two-component system; rr*-*sk*, *rr*-*sk* with a point mutation in *rr*.

b Abbreviations in antibiotics: AMP, ampicillin; CAZ, ceftazidime; CTX, cefotaxime; MER, meropenem; CIP, ciprofloxacin; KAN, kanamycin; TET, tetracycline.

c ND, not determined.

On the other hand, a disruption of the mutated RR-coding gene, the one causing the Glu166Lys mutation, in strain W36F2 abolished the high ceftazidime resistance (Table 1). However, introduction of an intact copy of the wild-type or the mutated RR-coding gene into this strain (i.e., 36F2Δrr*) restored the ceftazidime resistance through complementation (Table 1). Notably, the mutated RR-coding gene resulted in an approximately 2-fold higher MIC than that observed for the wild-type gene (Table 1), thus demonstrating the effect of this RR mutation on the ceftazidime resistance. A disruption of the SK-coding gene (i.e., BTH_RS09130) also abolished the antibiotic resistance (Table 1), demonstrating that the proper functioning of the mutated RR requires the intact SK.

The requirement of the RR and SK pair for inducing activity of PenL was also demonstrated for the case of wild-type PenL. Specifically, a disruption of the RR- or the SK-coding gene decreased the MIC for ampicillin, the digestible substrate for PenL. Introduction of the wild-type or the mutated RR-coding gene into the strain with the disrupted RR-coding gene (i.e., E264ΔRR) restored the MIC for ampicillin (Table 1). Furthermore, the mutations in the RR led to the abnormally high activity of the enzyme. Although phosphorylation of the RR or the SK or phosphate transfer from the SK to the RR were not analyzed, these MIC profiles suggest the protein pair forms a functional TCS that plays an essential role in the normal activity of (ESBL-) PenL. Considering TCSs are signal transduction systems that regulate the transcription of genes under their control (24, 25), the data suggest that TCS studied here is likely to be the regulator governing the expression of (ESBL-) PenL.

### The induction of *penL* by the TCS requires the associated *cis* elements.

**FIG 3:**
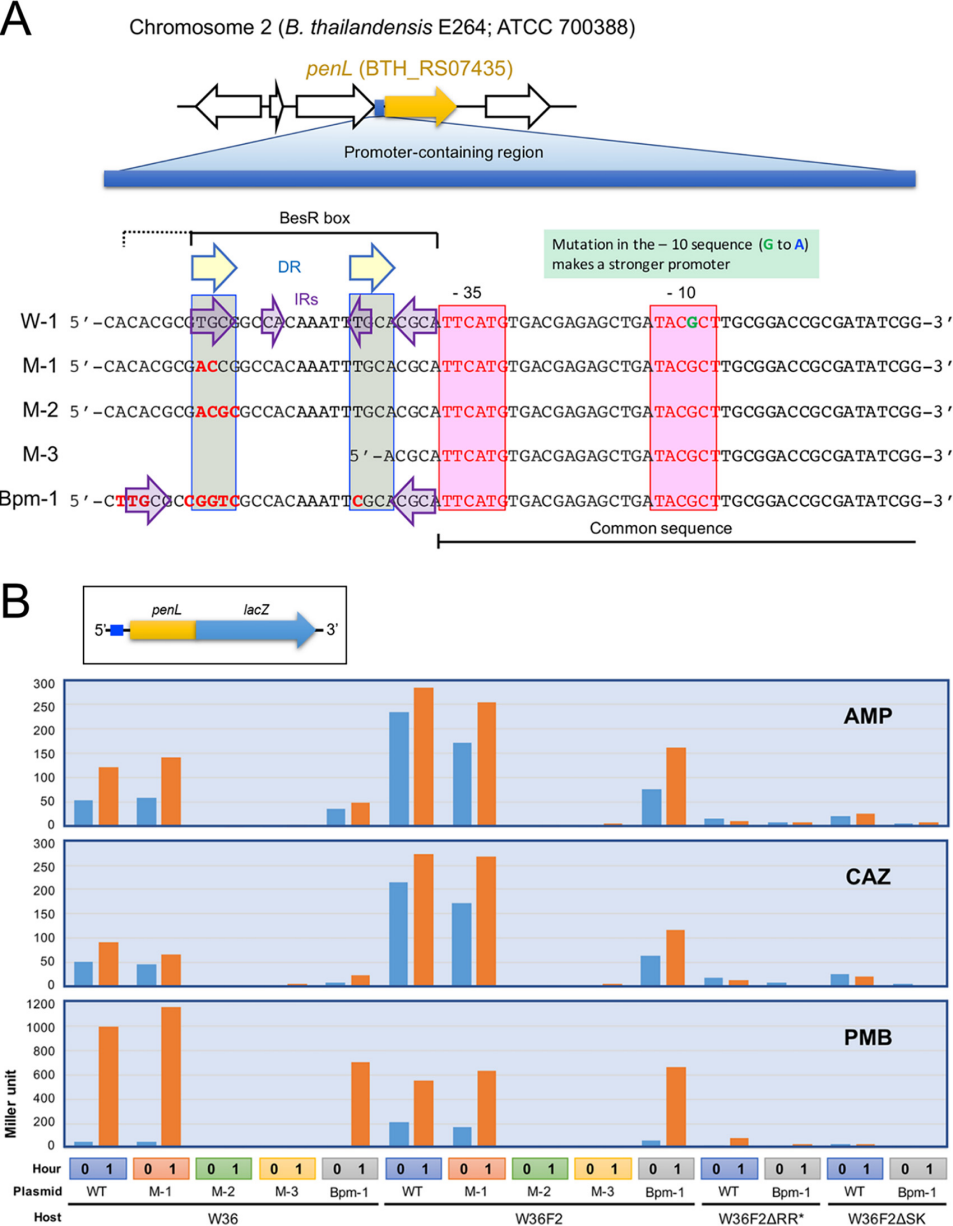
The *cis* elements present in the *penL* promoter and its involvement in gene expression. (A) The sequence of the promoter and the putative *cis* elements. The putative –10 and –35 sequences comprising the core of the promoter and the putative BesR-box are shown. The dotted line extending the BesR-box is drawn based on the “TGCG” sequence present in B. pseudomallei and B. mallei. The nucleotide “G” in the –10 sequence, previously reported to be substituted with “A” in the stronger promoter (17), is denoted in green. The wild-type (W-1) and *cis* element variants (M-1, M-2, and M-3) and Bpm-1 that were tested with the *penL*-*lacZ* reporter are shown. Bpm-1 is the corresponding region from B. pseudomallei and B. mallei, and it is identical in both species. (B) The effect of the variations in the *cis* element and the host background on *penL* expression. The *penL-lacZ* fusion constructed in a plasmid to report gene expression is shown at the top. The variants of the *cis* elements, shown above in A, were tested with the *penL-lacZ* reporter in different hosts. Strain W36 is strain E264 having ESBL-PenL, W36F2 is W36 having a hyperactive response regulator (RR*) with the Phe94Cys mutation, W36F2ΔRR is W36F2 lacking the RR*, and W36F2ΔSK is W36F2 lacking the sensor kinase (SK). Gene expression levels measured immediately before (0 h) and after (1 h) the antibiotic exposure, expressed in Miller units, are shown in the bar graph. Ampicillin (AMP), ceftazidime (CAZ), or polymyxin B (PMB) were used to induce the *penL*-*lacZ* reporter.

Gene expression regulation by a TCS is executed by the binding of the activated RR to *cis* elements associated with target genes (34). In the *penL* promoter, we found a region comprising the *cis* elements (5′-TGCGGCCACAAATTTGCACGCA-3′) present immediately upstream of the putative –35 sequence (Fig. 3A). This region contained two pairs of inverted repeats (IRs) and a direct repeat (DR). The first IR consisting of 4-bp sequences present at the leftmost and rightmost part of the region comprising the *cis* elements, and the second pair consisting of 2-bp sequences present at positions between the 4-bp sequences comprising the first IR, are “TGCG/CGCA” and “CA/TG,” respectively (Fig. 3A). The DR consists of a pair of 4-bp sequences that include a 1-bp mismatch, “TGCG” and “TGCA,” which occur at the leftmost part and before “CGCA” at the rightmost part of the region comprising the *cis* elements, respectively (Fig. 3A). The leftmost sequence “TGCG” is shared by the IR and DR. In E. coli, RstA binds to a DR of “TACA,” known as the “RstA-box” (35). The “TACA” sequence in the RstA-box resembles the “TGCG” sequence present in the *penL* promoter, as both “A” and “G” are purines. In Klebsiella pneumoniae, an RstA homologue has been shown to bind to the imperfect DR “TACA/TACT” (27). The “TGCG/TGCA” DR present in the *penL* promoter, also being an imperfect DR, and the mismatch also being at the fourth position, is an intriguing similarity between the *penL* promoter and the RstA homologue (Fig. 3A).

To test the involvement of the putative *cis* elements in *penL* gene expression, we constructed a *penL*-*lacZ* reporter gene fusion downstream to the wild-type or the variants of the putative *cis* elements (see Materials and Methods). The different types of upstream *cis* elements included: one with the intact sequence (control; W-1), one with two bases of the leftmost sequence “TGCG” changed (from TG to AC) (M-1), one that had all four bases in “TGCG” changed (from TGCG to ACGC) (M-2), and one that lacked most of the IRs and DR (M-3). The effects of these *cis* elements were compared in the presence of sublethal levels of three antibiotics (Fig. 3B), namely, ampicillin and ceftazidime, of which, the former is a digestible substrate of the wild-type PenL and the latter is a digestible substrate of ESBL-PenL, respectively, and polymyxin B, a non-β-lactam antibiotic, also known to disrupt the bacterial cell envelope (36). In the β-galactosidase assays that measured the expression of the *penL*-*lacZ* reporter linked to the wild-type *cis* elements (as in W-1), all three antibiotics induced *penL* expression; however, the induction by polymyxin B was significantly stronger than that by the other antibiotics (Fig. 3B). Notably, *penL* exhibited high basal-level expression and both basal and induced expression was significantly increased with the hyperactive response regulator (RR*) with the Phe94Cys mutation as shown in the W36F2 background (Fig. 3B). *penL* expression was severely affected when any of the TCS proteins was disrupted (Fig. 3B), and this result correlated with the decreased MICs of the TCS mutants for β-lactams as shown in Table 1. A change of two nucleotides, as in M-1, did not seriously affect *penL* expression; however, a change in all four nucleotides, as in M-2, considerably disrupted the *penL* expression (Fig. 3B). M-3, which lacked most of the putative *cis* element, did not exhibit a significant *penL* expression. Thus, the *cis* elements can be considered to have a role, most likely as an RR binding site, in *penL* induction by the TCS.

### The TCS-mediated β-lactamase gene regulatory system may also be functional in B. pseudomallei and B. mallei.

To determine if this regulation operates in species other than B. thailandensis, we tested the corresponding sequence, Bpm-1, from B. pseudomallei and B. mallei, which is identical in both species (Fig. 3A). Bpm-1 is slightly different from its B. thailandensis counterpart and does not contain the same DR or IR features (Fig. 3A). However, Bpm-1 has a characteristic “TGCG” sequence immediately upstream of this region, which makes a perfect IR pair with the “CGCA” sequence present downstream (Fig. 3A). We tested Bpm-1 by linking it to the *penL*-*lacZ* reporter, as was done with other variant *cis* elements (Fig. 3A). Despite deviations in the sequence from the B. thailandensis *cis* elements region, Bpm-1 successfully activated the *penL*-*lacZ* reporter, albeit at a slightly lower level compared with the activation in W-1 or M-1 (Fig. 3B). This weaker gene activation may be attributable to suboptimal interactions between Bpm-1 and the B. thailandensis RR, which is highly similar, but not identical, to the RRs from B. pseudomallei and B. mallei (96.61% identity at the AA-level) (Fig. 3B). Nevertheless, this result suggests this TCS-mediated β-lactamase gene regulatory system may be also functional in B. pseudomallei and B. mallei.

### Cell envelope disturbance triggers the TCS-mediated *penL* gene activation.

Among the antibiotics that activated the *penL-lacZ* reporter, ampicillin and ceftazidime are β-lactams; however, polymyxin B is not (Fig. 3B). Notably, this non-β-lactam, polymyxin B, was the strongest stimulant of the *penL-lacZ* reporter activation, suggesting the TCS did not detect β-lactam molecules, or cell wall fragments that would have been accumulated in the periplasm in the presence of β-lactams.

**FIG 4:**
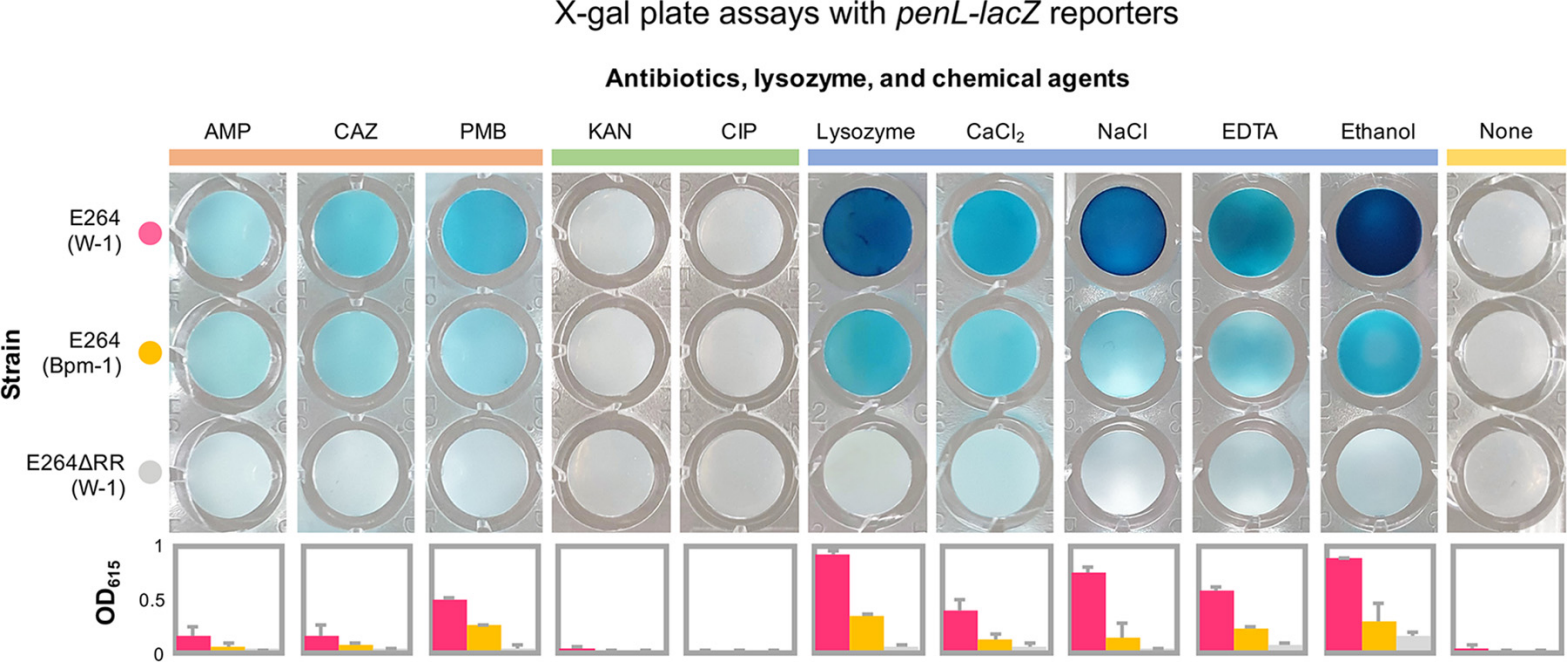
The X-gal plate assays with *penL-lacZ* reporters. The plate assays show the expression levels of the *penL* promoter in response to various antibiotics, lysozyme, and chemicals. Five antibiotics tested include the three that target the envelop, ampicillin (AMP), ceftazidime (CAZ), and polymyxin B (PMB), that were shown to activate the *penL* promoter (Fig. 3B) and two others, kanamycin (KAN) and ciprofloxacin (CIP), that do not directly disturb the membrane integrity. Lysozyme, CaCl_2_, NaCl, EDTA, and EtOH that also directly disrupt the envelope, are tested. Concentrations of the antibiotics, lysozyme, and chemicals are shown in Materials and Methods. B. thailandensis strains harbor a plasmid with the *penL*-*lacZ* reporter linked to the upstream BesR box from either B. thailandensis E264 (i.e., W-1) or B. pseudomallei and B. mallei (i.e., Bpm-1) (see Fig. 3A for W-1 and Bpm-1). Graphs in the last row show the quantified color intensities of the samples measured as OD_615_ (see Materials and Methods).

To test if cell envelope disruption results in *penL* expression, we conducted an X-gal plate assay with B. thailandensis strains harboring the *penL*-*lacZ* reporters (Fig. 4) (see Materials and Methods). While the known-inducers, ampicillin, ceftazidime, and polymyxin B (Fig. 3B), activated the *lacZ* reporters as expected, kanamycin and ciprofloxacin, which do not directly disturb the cell envelope, failed to do so (Fig. 4). Notably, the enzymes and chemical agents that are known to disturb the bacterial cell envelope, such as lysozyme (37), CaCl_2_ (38), NaCl (39), EDTA (40), or ethanol (40), also activated the *penL*-*lacZ* reporters (Fig. 4). Furthermore, the *penL*-*lacZ* gene activations by these treatments occurred only when *besR* was intact, like the treatments with ampicillin, ceftazidime, and polymyxin B (Fig. 4). Together, this X-gal plate assay demonstrated cell envelope disturbance triggers *penL* induction by means of the TCS. Based on this finding, we named the TCS, *Burkholderia* envelope stress response regulator and sensor kinase (BesRS), and the *cis* elements required for the gene induction, the BesR box (Fig. 3A).

### The BesRS regulon contains genes encoding stress response, antibiotic resistance, and β-lactam catabolism.

**FIG 5:**
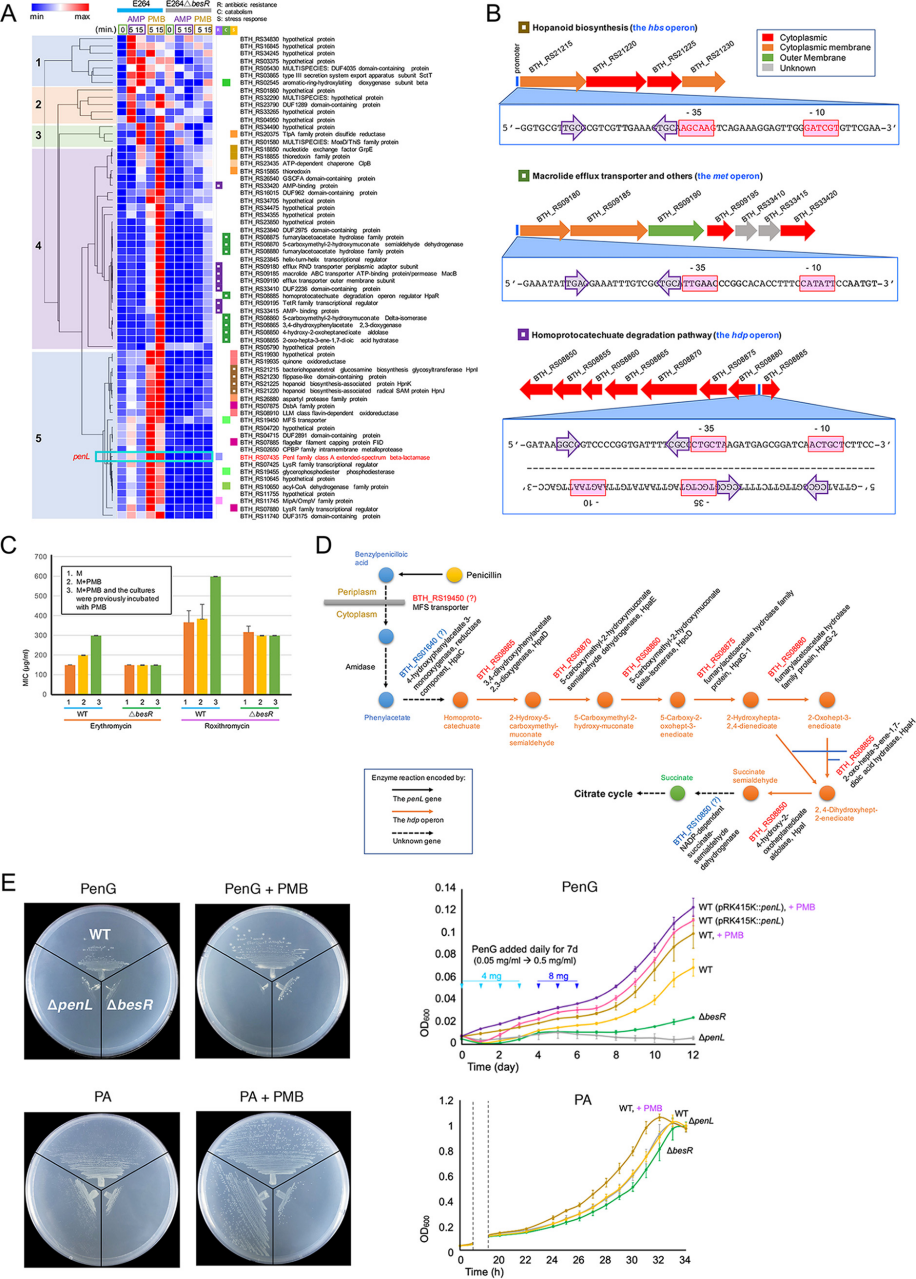
The BesRS regulon. (A) A heatmap constructed with the RNA-seq transcriptome data showing the genes that are induced in response to ampicillin (AMP) and/or polymyxin B (PMB) in the wild-type B. thailandensis E264 but not in the *besR*-null mutant. The RNA-seq data corresponding to this heatmap is shown in Table S1. Hierarchical clustering of the genes revealed five expression patterns. Most of the genes are predicted to have a role in antibiotic resistance (R), catabolism (C), or stress response (S), and they are denoted with a square before their locus tags. The squares associated with the genes comprising a putative operon are color-coded together, and they are further marked with a white box if the operon is displayed in B. The *penL* gene is highlighted in red and its expression pattern is denoted in a blue box. (B) The operons that may encode the hopanoid biosynthesis (the *hbs* operon), a macrolide efflux transporter (the *met* operon), or the homoprotocatechuate degradation pathway (the *hdp* operon) have putative *cis* elements that resemble the BesR box found in the *penL* promoter (Fig. 3A). (C) MICs for macrolides (M) (erythromycin and roxithromycin) of the wild-type (WT) and the *besR-*null mutant (Δ*besR*) of B. thailandensis E264. The bacterial cultures were grown with or without PMB (polymyxin) (50 μg/mL), and the MIC plates for an M were also with or without PMB to analyze the effect of PMB on gene induction. (D) A model for the penicillin catabolic pathway in B. thailandensis E264. The enzymes encoded by the *hdp* operon (BTH_RS09180 – BTH_RS08880) are responsible for the main steps of the sequential degradation of homoprotocatechuate into succinate. Additional genes, BTH_RS07435 (encoding *penL*), BTH_RS19450 (encoding an MFS transporter), BTH_RS01640 (encoding 4-hydroxyphenylacetate 3-monooxygenase, reductase component), and BTH_RS10850 (encoding succinate semialdehyde dehydrogenase), may be also involved in the complete catabolism of a β-lactam. The gene encoding the amidase has not been identified. (E) Growth of B. thailandensis strains on penicillin G (PenG) or phenylacetate (PA). The wild-type (WT), the *penL*-null mutant (Δ*penL*), and the *besR*-null mutant (Δ*besR*) of B. thailandensis E264 were streaked on AB agar plates supplemented with PenG (0.5 mg/mL) or PA (1.0 mg/mL) as the sole source of carbon and energy. The induction of the BesRS regulon by polymyxin B (PMB) (50 μg/mL) stimulated the growth of the wild-type strain on the PenG plate, and of both, the WT and the *penL*-null mutant, on the PA plate. The growth of the *besR*-null mutant was not stimulated by PMB on either plate. In liquid cultures with PenG, the WT exhibited slow growths reaching the OD_600_ of 0.07 in 12 days, but it grew better when the inducer of the BesRS regulon, PMB, was added. The *penL* mutant did not grow, and the *besR* mutant exhibited only weak growths. To help the strains grow with less antibiotic burden, PenG was added daily in small amounts up to 0.5 mg/mL for 7 days. WT (pRK415K::*penL*), which harbors multiple copies of *penL*, grew better than the WT and its growth was augmented by induction with PMB. On phenylacetate, all of the strains grew well, and the WT exhibited increased growths after induction with PMB.

To identify the BesRS regulon across the genome, we conducted an RNA-seq transcriptome analysis with the wild-type and the *besR* null mutant of B. thailandensis E264 after exposing them to sublethal levels of ampicillin (10 μg/mL) or polymyxin B (50 μg/mL) for 5 and 15 min (see Materials and Methods) (Fig. 5A). We found various genes to be significantly upregulated in response to antibiotic treatments in the wild-type strain, but not in the *besR*-null mutant (Fig. 5A; see Table S1 for the RNA-seq data). We failed to find any genes downregulated for the same treatments. Most of the BesRS-regulated genes had a predicted role in stress response, antibiotic resistance, or catabolism (Fig. 5A). Many of them were found to belong to a few operons that may be responsible for the major functions encoded by the BesRS regulon.

10.1128/mbio.01749-22.3 TABLE S1 The RNA-seq transcriptome data of the BesRS regulon. These data were used to generate the heatmap presented in Fig. 5A. Download Table S1, XLSX file, 0.02 MB. Copyright © 2022 Lee et al. 2022 Lee et al. https://creativecommons.org/licenses/by/4.0/ This content is distributed under the terms of the Creative Commons Attribution 4.0 International license.

Consistent with the β-galactosidase assay results (Fig. 3B), the basal-level expression of the β-lactamase gene *penL* was higher in the wild-type strain than that in the *besR*-null mutant, and its expression was increased in response to both ampicillin and polymyxin B, but more pronounced in response to polymyxin B (Fig. 5A). A previous study showed the *penL* ortholog in B. pseudomallei, *penA* (BPSS0946), can be cotranscribed with an upstream gene *nlpD1* (BPSS0945) as an operon (41). However, the *nlpD1* ortholog in B. thailandensis presented approximately 3- to 4-fold weaker expression than that of *penL*. Its expression was induced by polymyxin B, but not by ampicillin, and this induction was not abolished in the *besR*-null mutant (Fig. S1). This expression pattern indicates the *penL* promoter is the target site of BesR for the regulation of the *penL* gene. On the other hand, the *besRS* genes had higher expression in the wild-type than in the *besR*-null mutant, suggesting they may be autoregulated by BesR (Fig. S1). However, they were not significantly induced by ampicillin or polymyxin B (Fig. S1).

10.1128/mbio.01749-22.1 FIG S1 A heatmap showing the expression patterns of the genes that encode PenL, lipoprotein NlpD, BesS, and BesR. The gene that encodes NlpD shows much weaker expression than that of its downstream gene that encodes PenL. Its expression was induced by polymyxin B, but not by ampicillin, and this induction was not abolished in the *besR*-null mutant. The *besRS* genes show higher expression in the wild-type than in the *besR*-null mutant. However, they were not significantly induced by ampicillin or polymyxin B. Download FIG S1, TIF file, 2.9 MB. Copyright © 2022 Lee et al. 2022 Lee et al. https://creativecommons.org/licenses/by/4.0/ This content is distributed under the terms of the Creative Commons Attribution 4.0 International license.

A hierarchical clustering sorted the genes belonging to the BesRS regulon into five groups, with groups 4 and 5, showing strong responses to polymyxin B (PMB), being the largest (Fig. 5A). Many of these genes are likely to be activated by BesR directly, particularly those belonging to group 5 that show strong inductions at 5 min of PMB treatment. However, there may also be other genes that are indirectly regulated, such as those belonging to group 4 that show prime inductions at the later point, i.e., 15 min of PMB treatment, possibly by one of the following regulators encoded by the genes included in the BesRS regulon: BTH_RS23845, BTH_RS07425, BTH_RS07880, BTH_RS09195, and BTH_RS08885 (Fig. 5A). Among the operons, those putatively encoding the hopanoid biosynthesis, a macrolide efflux transporter, and the homoprotocatechuate degradation pathway, which we call the *hbs*, *met*, and *hdp* operon, respectively, were the most notable due to their large sizes and the distinct functions that they encoded (Fig. 5B). Intriguingly, these operons have a perfect/imperfect IR or DR pair at their 5′-ends (Fig. 5B) resembling the BesR box in the *penL* promoter (Fig. 3A), although further experimental verifications to confirm this are needed.

The *hbs* operon may be important for survival under antibiotic-induced stress (Fig. 5B). Hopanoid lipids have been shown to facilitate bacterial survival under stress by lowering the fluidity and permeability of membranes to prevent leakage of cations and proteins across the membrane (41, 42). Hopanoids also have been reported to affect the action of membrane-associated proteins, which turned out to be critical for the function of a multidrug efflux system in Methylobacterium extorquens (43). Genes encoding thioredoxin-related proteins (i.e., BTH_RS15865, BTH_RS18855, and BTH_RS07875) may also have a role in survival under stress (Fig. 5A). Thioredoxin has been associated with oxidative stress response and protein repair (44). Other stress-response genes include BTH_RS18850, which codes for the nucleotide exchange factor GrpE, and BTH_RS23435, which codes for the ATP-dependent chaperone ClpB (Fig. 5A). GrpE is reported to cooperate with the heat shock protein DnaK in refolding denatured proteins, and ClpB is reported to cooperate with DnaK and GrpE (45, 46). Three genes in the *met* operon, BTH_RS09180, BTH_RS09185, and BTH_RS09190 (Fig. 5B), may encode a MacA-MacB-TolC tripartite complex spanning the cytoplasmic and the outer membrane as found in many pathogens (47) (Fig. 5B). This efflux system is known to actively extrude various substrates, including macrolides, virulence factors, peptides, and cell envelope precursors (47). The MICs of the wild-type B. thailandensis E264 for two macrolides tested, erythromycin and roxithromycin, showed a concordant increase in the presence of polymyxin B, but those of the *besR*-null mutant did not (Fig. 5C). Further increase in the MICs was observed on adding polymyxin B to the cells earlier, as they were being grown before the test. This suggests the induction levels of the efflux transporter may have affected these MICs. The BesRS regulon also includes BTH_RS11745, which encodes an antibiotic-resistance-associated MipA/OmpV family protein (Fig. 5A). MipA in E. coli is an outer membrane protein associated with increased or decreased resistance to various antibiotics (48). Tests with antibiotics revealed the wild-type B. thailandensis exhibited increased resistance to chloramphenicol and ciprofloxacin when induced with polymyxin B, but the *besR*-null mutant did not (Fig. S2). However, more studies are needed to conclusively link the observed resistance phenotypes to the MipA/OmpV family protein. Intriguingly, seven genes that may encode the homoprotocatechuate degradation pathway have been found in the BesRS regulon (Fig. 5B and D). The gene content of this *hdp* operon is similar to that of the *hpaGEDFHI* cluster found in Burkholderia xenovorans LB400 (49). The homoprotocatechuate degradation pathway encoded by this gene cluster is one of the central pathways involved in breaking down the products of various peripheral pathways associated with processing a broad range of aromatic compounds (41, 50). Recently, penicillins have been shown to be catabolized by soil isolates of a *Burkholderia* sp. and of several other bacterial species (22). In these soil isolates, genes encoding β-lactamase, amidase, phenylacetic acid catabolon, and major facilitator superfamily (MFS) transporter have been associated with β-lactam catabolism (22). Based on this finding, we propose the following pathway for β-lactam catabolism in B. thailandensis (Fig. 5D). First, penicillin molecules are hydrolyzed by the β-lactamase PenL in the periplasm to produce benzylpenicilloic acid, which might be translocated by an MFS transporter (possibly encoded by BTH_RS19450) across the cytoplasmic membrane. This is followed by the possible release of phenylacetate from this benzylpenicilloic acid due to the action of an amidase (the gene for this enzyme is not known). The benzyl ring of the phenylacetate might then be activated by hydroxylation reactions (possibly encoded by BTH_RS01640) to yield homoprotocatechuate. This primary substrate could then be sequentially degraded into succinate by the enzymes encoded by the seven genes in the *hdp* operon. Succinate might then be routed to the citrate cycle in the form of succinyl-CoA (Fig. 5D). Concordantly, B. thailandensis E264 grew on solid media with penicillin G or phenylacetate as the sole source of carbon and energy, and this growth was significantly augmented with the addition of polymyxin B (50 μg/mL), possibly due to the induction of the *hdp* operon and other genes belonging to the BesRS regulon (Fig. 5E). Overall, the growth of B. thailandensis strain E264 appeared more pronounced on the media with phenylacetate than on the media with penicillin G. As expected, the growth on penicillin G, but not on phenylacetate, was affected by a mutation in *penL*, and the induction of the growth by polymyxin B on both substrates was affected by a null mutation in *besR* (Fig. 5E). In liquid media with penicillin G (0.5 mg/mL) as a sole source of carbon and energy, B. thailandensis E264 grew slower than on solid media, taking as long as 12 days to reach the OD_600_ of 0.1 from 0.01, but it grew better with induction of polymyxin B (Fig. 5E). The *penL* mutant did not grow, and *besR* mutant exhibited only weak growths (Fig. 5E). To test if B. thailandensis E264 grows better when *penL* expression is significantly increased, we transfected the wild-type with pRK415K::*penL* to increase the copy number of *penL*. As expected, this strain grew better than the strain without the extra *penL* copies and the growth was further augmented when polymyxin B was added, demonstrating the significant role played by the first enzyme PenL in the penicillin G catabolism (Fig. 5D). *Burkholderia* soil isolate selected based on its ability to grow on media with penicillin G also presented slow growth on media with penicillin G (22). However, this soil isolate grew slightly faster than B. thailandensis E264 reaching the OD_600_ of 0.1 within a week (22). On the other hand, B. thailandensis E264 grew well on phenylacetate (1 mg/mL), reaching the OD_600_ of 1.0 from 0.01 in approximately 30 h (Fig. 5E). On phenylacetate, the wild-type and *penL* mutant grew better than the *besR* mutant, and the growth of the wild-type (and the *penL* mutant) (data not shown) was augmented by polymyxin B (50 μg/mL) (Fig. 5E).

10.1128/mbio.01749-22.2 FIG S2 MICs for chloramphenicol and ciprofloxacin of the wild-type (WT) and the *besR-*null mutant (Δ*besR*) of B. thailandensis E264. The wild-type exhibited increased resistance to the antibiotics when induced with polymyxin B (PMB), but the *besR*-null mutant did not. The bacterial cultures were grown with or without PMB (50 μg/mL), and the MIC plates for an antibiotic (A) also contained or lacked PMB. Download FIG S2, TIF file, 2.5 MB. Copyright © 2022 Lee et al. 2022 Lee et al. https://creativecommons.org/licenses/by/4.0/ This content is distributed under the terms of the Creative Commons Attribution 4.0 International license.

## DISCUSSION

**FIG 6:**
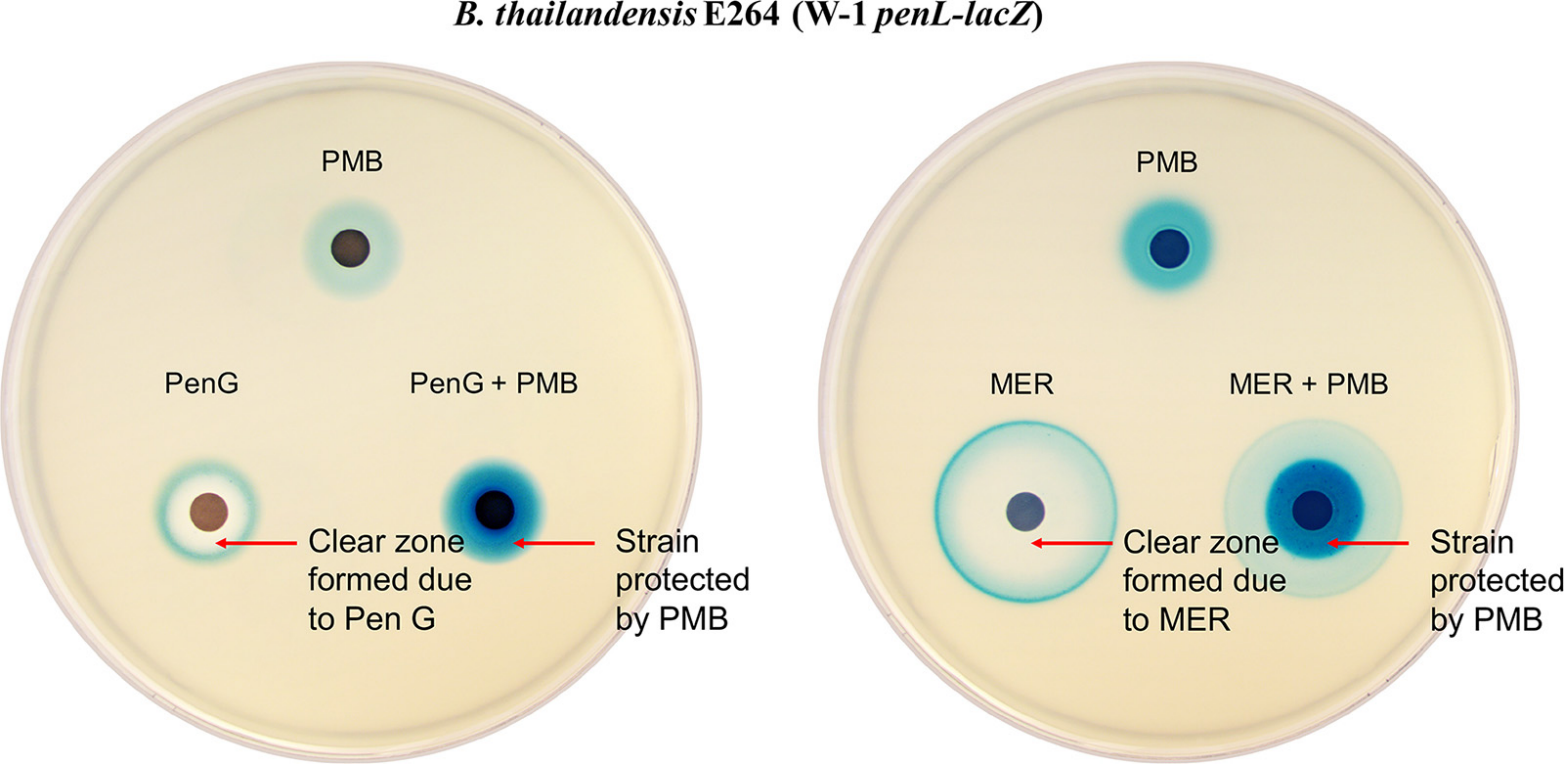
Antibiotic disc plate assay. The assay plates contain the ABG minimal medium with X-gal, and B. thailandensis strain E264 harboring a plasmid with the W-1 *penL*-*lacZ* reporter was overlaid in soft agar on top of the plate. Paper discs contain antibiotics (PMB, polymyxin B; PenG, penicillin G; MER, meropenem). Addition of polymyxin B to the discs containing penicillin G or meropenem results in better protection of B. thailandensis from the action of these antibiotics in areas where the cells would have succumbed to it. Blue color of X-gal shows the induction of *penL* probed with the W-1 *penL*-*lacZ* reporter.

Unlike mechanisms sensing cell wall fragments liberated by β-lactams using an AmpR-type regulator or a TCS (1, 3, 5, 9), or detecting β-lactam molecules directly with a TCS (10), this newly characterized TCS-governed β-lactamase gene regulation mechanism that responds to disruptions in the cell envelope may not seem sophisticated, or even adequate, particularly in the absence of β-lactams. However, as part of a regulon encoding functions for survival, including multiple antibiotic resistances, stress responses, and β-lactam catabolism (Fig. 5A), this β-lactamase gene regulation mechanism may play a pivotal role in facilitating bacterial growth in the soil environment, which is highly competitive due to the presence of multiple antibiotic-producing bacteria and fungi (21, 23). Antibiotic disc assays with β-lactam antibiotics, penicillin G and meropenem, support this notion (Fig. 6). Addition of polymyxin B to the discs containing penicillin G or meropenem led to the wild-type B. thailandensis being better protected from the action of both antibiotics in areas where the cells would have succumbed to it (Fig. 6). In these areas of the plates, expression of *penL* probed with the *penL*-*lacZ* reporter showed strong induction by polymyxin B (Fig. 6). Such cross-protection conferred by the response to cell envelope damage may have evolved in the *Burkholderia* lineage in their soil niches. Notably, CpxAR, a TCS closely related to BesRS, has been shown to contribute to the colonization and survival of Salmonella enterica serovar Typhimurium in the gut environment (51).

β-Lactams and polymyxin B have different targets on the bacterial envelope, and therefore, cause different types of damages. While β-lactams disturb the biosynthesis of peptidoglycan by inhibiting DD-transpeptidases, also known as penicillin-binding proteins (PBPs) (41), thereby releasing muropeptides that may induce β-lactamase gene expression (1, 3, 4), polymyxin B disrupts the outer and inner membranes of Gram-negative bacteria by binding to lipid A of the lipopolysaccharide (LPS) (31, 36). Therefore, the signals detected by BesRS from disturbances by β-lactams and polymyxin B may be different. Notably, polymyxin B triggers BesRS stronger than β-lactams, despite it not being an effective antibiotic for *Burkholderia* spp. (52). This suggests BesRS may be capable of sensing a subtle, transient disturbance in the cell envelope. CpxARs from E. coli and S. enterica serovar Typhimurium have also been reported to be activated by polymyxin B (41, 51).

The BesRS-mediated gene regulation in B. thailandensis, reported in the present study, might be also functional in B. pseudomallei and B. mallei. This can be inferred from the results showing the putative *cis* element in these species also led to the regulation of β-lactamase expression when tested with the *penL*-*lacZ* reporter (Fig. 3). BesRS being essential to the induction of PenI-type class A β-lactamase expression makes it an attractive target for the development of combination therapies for highly effective antibiotic regimens against *Burkholderia* infections, similar to that proposed for TCSs in general with regard to treatment of for pathogenic bacterial infections (53).

## MATERIALS AND METHODS

### Bacterial strains and cultures.

E. coli was grown in LB, and B. thailandensis strains were grown in LB or *Agrobacterium* (AB) minimal media containing 0.25% glucose (ABG) (54), at 37°C. For E. coli, 100 μg/mL ampicillin, 10 μg/mL tetracycline, and 50 μg/mL kanamycin, and for B. thailandensis E264, 50 μg/mL tetracycline and 250 μg/mL kanamycin was used with cloning vectors.

### Selection of bacterial mutants highly resistant to ceftazidime.

In a previous study, we have performed selection experiments with B. thailandensis E264 (MIC for ceftazidime: 1 μg/mL) in the presence of 5 μg/mL of ceftazidime, and obtained 29 *penL* mutants (17). Among these, two mutants, W35 and W36, having Asp179Asn (MIC for ceftazidime: 80 μg/mL) and Glu166Lys (MIC for ceftazidime: 49 μg/mL) in PenL, respectively (17), were used for selection experiments with 100 μg/mL ceftazidime. A single colony of W35 or W36 was grown overnight in 2 mL of LB broth at 37°C with shaking at 250 rpm. Overnight cultures were pelleted and resuspended in 2 mL of fresh LB broth to yield approximately 5 to 8 × 10^9^ CFU/mL. A total of 100 μL of cell suspension was spread on LB agar plates containing 100 μg/mL ceftazidime, which were incubated at 37°C for 48 h or until colonies were visible.

### Sequence analysis of the bacterial isolates highly resistant to ceftazidime.

Genomic DNA was purified from bacterial isolates using the Wizard Genomic DNA purification kit (Promega, Madison, WI, USA). Before sequencing the genomic DNA samples, we first checked the sequence of the *penL* promoter region in each isolate to find the mutants that have the previously identified promoter mutation (i.e., G to A in the –10 sequence) (17). Genomic DNA samples from the isolates that did not have this promoter mutation were sequenced using HiSeq 2000 (Illumina, San Diego, CA, USA) at Macrogen Inc. (Seoul, South Korea). A DNA library of genomic DNA fragments was prepared according to the Illumina protocols; library quality was validated using the Agilent 2100 Bioanalyzer (Agilent Technologies, Santa Clara, CA, USA). Sequences were read using the 101-bp paired-end reading module and data were analyzed using the CLC Genomics Workbench software (Qiagen, Redwood City, CA, USA). Briefly, raw sequence data were paired-trimmed. The resulting reads were mapped to B. thailandensis E264 (Accession number: CM000438.1 and CM000439.1) reference genome to detect SNPs and deletion/insertion polymorphisms.

### Determination of MICs.

MICs for antibiotics were measured according to the protocol described in a previous study (17). The series of agar plates used contained antibiotics at successively increasing concentrations with 2-fold increments starting from 0.5 or 1 μg/mL. Average values were calculated based on the results of three independent experiments.

### Three-dimensional (3D) modeling of B. thailandensis BesR (BTH_RS09135).

The structure of B. thailandensis BesR was simulated using the X-ray structure of the homologous RstA of Klebsiella pneumoniae (27) (PDBID: 4NIC) as a template (sequence identity, 44.2%) using Modeller (55). After generating the wild-type structure of B. thailandensis BesR, mutant structures were generated using Chimera (56). Molecular dynamics simulations with explicit water molecules were performed on wild-type and mutant BesRs using NAMD (57) with CHARMM36 force field (58). The simulation was performed for a total of 100 ns and results from the last 30 ns were used.

### Generation of null mutations in specific genes.

To disrupt the *besR* gene (BTH_RS09135), a 1,248-bp amplicon (362 bp upstream of the start codon to 175 bp downstream of the stop codon) was obtained by PCR using besR-KF (5′-ATATATGGTACCGCGTTCGAGAGCTGGTATTT-3′) and besR-KR (5′-ATATATGGTACCGAAAGTCGCATTGCGTCAC-3′), containing the KpnI recognition site (underlined). The PCR product was digested with KpnI; the fragment was ligated into pUC19, which was digested with KpnI and treated with calf intestinal alkaline phosphatase (CIP) (NEB, Ipswich, MA, USA). The resulting pUC19::*besR* construct was double-digested with BseRI and PflFI and blunt-ended with T4 DNA polymerase to remove an internal region, from position 6 to 220, from the 711-bp-long *besR* coding sequence. The fragment was ligated with a previously prepared tet^r^ cassette (17); the pUC19::*besR*::tet^r^ construct, containing *besR* disrupted by the tet^r^ cassette, was used to transform B. thailandensis E264 and W36F2 to obtain *besR*-null mutants using a natural transformation method described in a previous study with some modifications (17). Allele exchange in the *besR*-null mutant was verified by PCR using besR_LF (5′-TGTATTCGGGGATGTCGTCT-3′) and besR_LR (5′-CGTTTCATCACGTCGAACC-3′), which bound to genomic regions outside *besR*.

To disrupt the *besS* gene (BTH_RS09130), a 1,550-bp amplicon (359 bp upstream of the start codon to 75 bp downstream of the stop codon) was obtained by PCR using besS-KF (5′-ATATATGGTACCGAACGCTTGGGAGTAAGCTG-3′) and besS-KR (5′-ATATATGGTACCCGGATTGAAAGCGCGAAT-3′), containing the KpnI recognition site (underlined). The PCR product was digested with KpnI and ligated into pUC19, as described above. The resulting pUC19::*besS* construct was double-digested with PflFI and NotI and blunt-ended with T4 DNA polymerase to remove an internal region, from position 99 to 363, from the 1,116 bp-long *besR* coding sequence. The resulting fragment was ligated with a tet^r^ cassette, as described above, and the pUC19::*besS*::tet^r^ construct, containing *besS* disrupted by the tet^r^ cassette, was used to transform B. thailandensis E264, W36F2, and W36F2-KX to obtain *besS* null mutants using a transformation method described in a previous study with some modifications (17). Allele exchange in *besS*-null mutants was verified by PCR using besS_LF (5′-CAGCGGATCAAGACCATTC-3′) and besR_LR (5′-CGCTCAAA CAAAGCTACACG-3′), which bound to genomic regions outside *besS*. Disruption of *penL* (BTH_RS07435), when it was called *penA*, has previously been described (17).

### Complementation of null mutations with copies of functional genes.

Wild-type and mutant *besR* PCR products (1,248 bp long, 362 bp upstream of the start codon to 175 bp downstream of the stop codon) were prepared, as described in the previous section, using genomic DNA from E264 and W36F2 as templates, respectively. The PCR products were digested with KpnI and ligated with pRK415K, a broad-host-range plasmid (59), treated with KpnI and calf-intestinal alkaline phosphatase-treated. The plasmid was then used to transform the E. coli strain S17-1 (60) using a conventional transformation method (61). The transformed S17-1 strain was conjugated with B. thailandensis E264 on ABG agar plates, containing 250 μg/mL kanamycin. The plates were incubated at 37°C for 2 days to select for transconjugants. Successful conjugation was confirmed by purifying the plasmid and analyzing the characteristic restriction patterns of the plasmid.

Wild-type and mutant *Burkholderia* envelope stress response regulator and sensor kinase (*besRS*) transconjugants were prepared, as described above, except the PCR product was amplified using besR-KF (5′-ATATATGGTACCGCGTTCGAGAGCTGGTATTT-3′) and besS-KR (5′-ATATATGGTACCCGGATTGAAAGCGCGAAT-3′) and E264 and W36F2 genomic DNA as the template. The 2,607-bp amplicon spanned from 362 bp upstream of the *besR* start codon to 75 bp downstream of the *besS* stop codon. E. coli S17-1 harboring pRK415K::*besRS* was conjugated with W36F2Δ*besR* to obtain transconjugants.

### Construction of the wild-type and variant *cis* elements linked to the *penL*-*lacZ* reporter.

To test putative *cis* elements that might interact with BesR, we generated DNA fragments with 5′-ends differing from those of the region upstream of the putative –35 sequence in the *penL* promoter: (i) W-1, which had the entire sequence of *cis* elements (5′-end of the fragment extended by 30 bp from the –35 sequence); (ii) M-1, which was of the same length as W-1, but had two substitutions (TG to AC) at the left repeat of the *cis* elements; (iii) M-2, which had two additional substitutions on the M-1 sequence (TGCG to ACGC); and (iv) M-3, which lacked a majority of the *cis* elements (5′-end extended by only 5 bp from the –35 sequence). The W-1, M-1, M-2, and M-3 fragments were generated using the forward primers penL-WTF (5′-ATATATGGTACCACACGCGTGCGGCCACAA-3′), penL-M-1F (5′-ATATATGGTACCACACGCG**AC**CGGCCACAA-3′), penL-M-2F (5′-ATATATGGTACCACACGCG**ACGC**GCCACAA-3′) (inserted substitutions are indicated in bold), and penL-M-3F (5′-ATATATGGTACCACGCATTCATGTGACGAGAG-3′), respectively, with a common reverse primer penL-RK (5′-ATATATGGTACCGCCGTTATCGCACCTTTATC-3′). The PCR products were digested with KpnI and cloned into pRK415K. To construct the Bpm-1 fragment with the *cis* elements from B. pseudomallei and B. mallei, the forward primer penA-Bp-BmF (5′-ATATATGGTACC**TTG**CGC**CGGTC**GCCACAAATT**C**GCACGCATTC-3′) (bases specific to these two species are indicated in bold) was used. The same reverse primer penL-RK and the B. thailandensis genomic DNA template were used for the PCR; therefore, the rest of the sequence that was amplified, besides the *cis* elements, is from B. thailandensis.

The *penL*-*lacZ* reporter fusion with one of the upstream fragments was constructed as follows: pRK415K, containing the *penL* fragment with an upstream region, was digested with KpnI and PstI. The *lacZ* cassette was obtained by digesting pLKC480 (62) with PstI and XbaI. Thereafter, the two DNA fragments were ligated with pRK415K that had been digested with KpnI and XbaI. The ligation mixture was transformed into E. coli S17-1, and the correct construct was selected and confirmed. E. coli S17-1 strains carrying pRK415K, containing various upstream regions linked to the *penL*-*lacZ* reporter, were mated with B. thailandensis strains to transfer the plasmids, as described above.

### The β-galactosidase assay with *penL*-*lacZ* reporters.

B. thailandensis strains harboring a *penL-lacZ* reporter plasmid were grown until the midexponential phase (OD_600_ of ≈ 0.6) in 30 mL ABG. Ampicillin, ceftazidime, or polymyxin B was added to a final concentration of 3, 10, or 50 μg/mL, respectively. Samples (1 mL) were collected at 0 or 1 h after the antibiotic treatments and incubated on ice for 10 min. Then, the cells were harvested and resuspended in 1.1 mL of Z-buffer (60 mM Na_2_HPO_4_, 40 mM NaH_2_PO_4_, 10 mM KCl, 1 mM MgSO_4_, and 0.27% [vol/vol] β-mercaptoethanol; pH 7.0). A cell suspension of 100 μL was taken for OD_600_ measurement, and the rest (1 mL) were treated with 100 μL of chloroform and 50 μL of 0.1% SDS for 5 min. Subsequently, the cell suspension was treated with ortho-nitrophenyl-β-d-galactopyranoside (0.4% [wt/vol] in Z-buffer) until sufficient yellow color developed and the incubation time was recorded. The reaction was stopped using 500 μL of 1 M Na_2_CO_3_ and the reaction mixture was centrifuged at 7,000 × *g* for 5 min. The supernatant (100 μL) was used for OD_420_ measurement. OD_600_ and OD_420_ were measured using the SpectraMax M2 microplate reader (Molecular Devices, Sunnyvale, CA, USA). β-Galactosidase activity was expressed in Miller units, as described by Schaefer et al. (63).

### The X-gal plate assays with *penL*-*lacZ* reporters.

The X-gal plate assays were conducted with the wild-type B. thailandensis E264 strain harboring the W-1 *penL-lacZ* reporter or the Bpm-1 reporter (see Fig. 3A), and the *besR*-null mutant strain harboring the W-1 *penL-lacZ* reporter (Fig. 4). These strains were grown overnight in 2 mL ABG medium containing kanamycin (250 μg/mL) with shaking at 37°C. Samples (100 μL) were taken from these cultures and diluted to a 1:100 concentration into fresh 10 mL ABG medium containing kanamycin (250 μg/mL) and were incubated with shaking until the cultures reached the mid-log-phase (OD_600_ = 0.6). Then, these cultures were pelleted by centrifugation at 4,000 × *g* for 10 min at 4°C, and the cells were resuspended in fresh 5 mL ABG medium containing X-gal (1 mg/mL). A 100-μL sample from each cell suspension was transferred into the wells of a 96-well plate for the addition of 100 μL of a solution containing an antibiotic, lysozyme, or a chemical agent as described below.

To analyze the response of the *penL-lacZ* reporters to antibiotics, lysozyme, and chemical agents, solutions were prepared in 100 μL of ABG medium and were added to the wells of the 96-well plate and mixed with the same volume of the bacterial suspensions containing X-gal prepared as described above. The final concentrations of the antibiotics in the mixtures were: 1,000 μg/mL for ampicillin, 60 μg/mL for ceftazidime, 25 μg/mL for polymyxin B, 100 μg/mL for kanamycin, and 0.05 μg/mL for ciprofloxacin. The final concentrations of lysozyme and the chemicals were: 10 mg/mL for lysozyme, 0.4 M for CaCl_2_, 0.6 M for NaCl, 7.5 mM for EDTA, and 10% for EtOH. The cell mixtures were incubated at 37°C until an observable color developed. The blue precipitates formed in the assays were quantified by recording OD_615_ with SpectraMax M2 microplate reader (Molecular Devices, Sunnyvale, CA, USA) that reflects both blue precipitates and cells, and then subtracts from these OD_615_ values the control sample values that reflect only the cells. The control samples were prepared simultaneously with the same cell suspensions without X-gal. Triplicate assays were used to generate the expression data shown in the graphs.

### RNA-seq transcriptome analysis.

Bacterial cultures of B. thailandensis E264 and its *besR* null mutant were incubated in LB medium in a shaking incubator at 37°C until the OD_600_ reached 0.6. At this point, 1 mL of the cultures were withdrawn as 0 min samples and were mixed with 2 mL of RNAprotect bacteria reagent (Qiagen Sciences Inc., Germantown, MD, USA). Subsequently, ampicillin or polymyxin B were added at a final concentration of 10 μg/mL and 50 μg/mL, respectively. Samples of 1 mL each were withdrawn from the cultures after 5 and 15 min and were immediately mixed with two volumes of RNAprotect bacteria reagent. The mixtures were centrifuged at 5,000 × *g* for 10 min and the supernatants were discarded. Total RNA was extracted from each of the cell pellets using an RNeasy minikit (Qiagen Sciences Inc., Germantown, MD, USA), according to the manufacturer's instructions. The total RNA samples were subjected to RNA-seq analysis at DNA Link Inc. (Seoul, South Korea). The purity of RNA was determined by assaying 1 μL of total RNA extract on a NanoDrop 8000 spectrophotometer (Thermo Fisher Scientific, Waltham, MA, USA.). The integrity of RNA was checked using an Agilent 2100 Bioanalyzer (Agilent Technologies, Santa Clara, CA, USA), expressed as an RNA Integrity Number (RIN) value. Total RNA sequencing libraries were prepared according to the manufacturer’s instructions for the Illumina TruSeq Stranded Total RNA sample prep kit with a Ribo-Zero Plus rRNA Depletion kit (Illumina, Catalog no. 20037135). The quality of the amplified libraries was verified by automated electrophoresis (TapeStation, Agilent). RNA-seq was performed using an Illumina NovaSeq 6000 system following provided protocols for 2 × 100 sequencing.

Raw RNA-seq reads were assembled into transcripts and their relative abundances were estimated using the tool Cufflinks (http://cole-trapnell-lab.github.io/cufflinks/). For the normalization of the transcript data from each experiment across all experiments, the default option, geometric, was used. Genes selected for analysis were clustered based on their expression patterns using a web-based tool, Morpheus (https://software.broadinstitute.org/GENE-E/).

### Bacterial growth on penicillin G or phenylacetate.

Single colonies of the wild-type, *besR*-null mutant, and *penL*-null mutant of B. thailandensis E264 were streaked on AB agar plates supplemented with penicillin G (0.5 mg/mL) (P3032; Sigma-Aldrich, St. Louis, MO, USA) or phenylacetate (1 mg/mL) (P16621; Sigma-Aldrich, St. Louis, MO, USA) and the plates were incubated at 37°C to test their ability to grow on these substrates on solid media. For liquid cultures, single colonies of the strains were used to inoculate 2 mL of ABG medium, and the cultures were grown to the late log phase (OD_600_ of about 0.8). These cultures (5 mL) were used to inoculate 80 mL of AB medium supplemented with penicillin G (final concentration: 0.5 mg/mL) or phenylacetate (1 mg/mL) and were incubated with shaking at 37°C. Instead of applying a high dose of penicillin G to the cells all at once, the antibiotic level was gradually increased daily from 0.05 mg/mL on the first day to 0.5 mg/mL after a week (see Fig. 5E). For the induction of the BesRS regulon, polymyxin B (50 μg/mL) was used.

### The antibiotics inhibition disc assay with penicillin G, meropenem, and polymyxin B.

Single colonies of each strain harboring the plasmid with the W-1 *penL-lacZ* reporter (Fig. 3A) were grown overnight in 2 mL ABG containing 250 μg/mL of kanamycin in a shaking incubator at 37°C. The overnight culture was pelleted by centrifugation at 4,000 × *g* for 10 min at 4°C, and the cells were resuspended in 2 mL of fresh ABG. The cells of 200 μL were mixed with 3 mL of 0.7% agar and were overlaid on top of the ABG agar plate supplemented with 500 μg/mL of X-gal. The antibiotic discs prepared by spotting and drying 5 μL of penicillin G (100 mg/mL) or meropenem (5 mg/mL) with or without polymyxin B (50 mg/mL) were placed on the plates. The plates were incubated overnight or until inhibition zones and X-gal color reaction were visible.
