# Supplementary figures and images for: A Two-Component-System-Governed Regulon That Includes a β-Lactamase Gene is Responsive to Cell Envelope Disturbance
Source: mBio. 2022 Aug 15;13(4):e01749-22. doi: 10.1128/mbio.01749-22 (PMC9426598; doi:10.1128/mbio.01749-22)

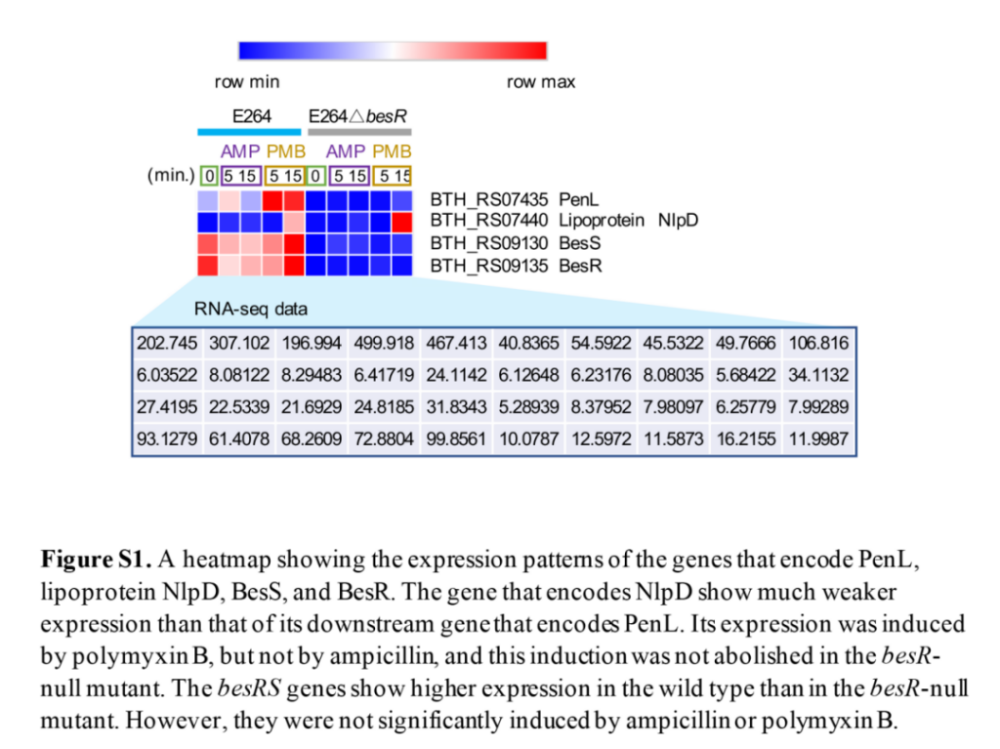

Supplement: FIG S1 [file mbio.01749-22-s0001.tif]

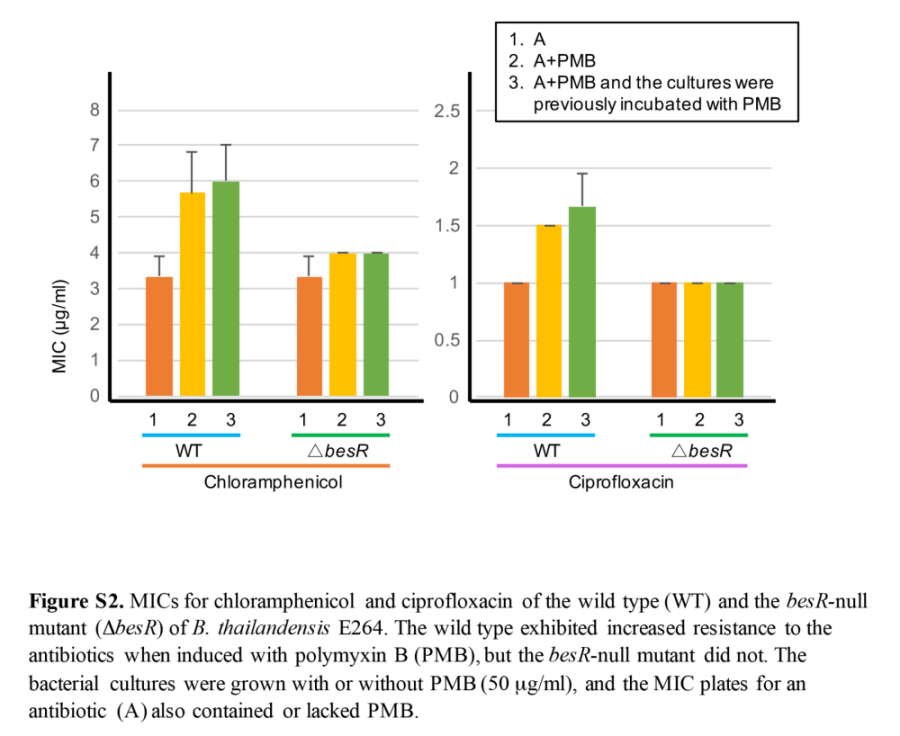

Supplement: FIG S2 [file mbio.01749-22-s0002.tif]
